# Molecular Transmission Network and Pretreatment Drug Resistance of Newly Diagnosed HIV-1 Infections in Taizhou, a Coastal City in Eastern China, from 2021–2023

**DOI:** 10.3390/pathogens14101030

**Published:** 2025-10-11

**Authors:** Junxiao Lin, Haijiang Lin, Guixia Li, Shanling Wang, Tingting Wang, Qiguo Meng, Tingting Hua, Yali Xie, Jiafeng Zhang, Weiwei Shen

**Affiliations:** 1 Taizhou Municipal Center for Disease Control and Prevention, Taizhou Municipal Institute of Health Supervision, No 4123, Zhongxin Avenue, Taizhou 318000, China; adtzhappy@163.com (J.L.); tzcdclhj@126.com (H.L.); lgx37130792@163.com (G.L.); wslwho@126.com (S.W.); wja_wdt@163.com (T.W.); mengqiguo601548652@163.com (Q.M.); 15256980828@163.com (T.H.); ylxie123@163.com (Y.X.); 2 Department of HIV/AIDS Control and Prevention, Zhejiang Provincial Center for Disease Control and Prevention, No. 3399, Binsheng Road, Hangzhou 310051, China

**Keywords:** HIV-1, CRF08_BC, molecular transmission network, pretreatment drug resistance

## Abstract

Objective: This study conducted a comprehensive analysis of molecular transmission networks and pretreatment drug resistance (PDR) in newly diagnosed HIV-1 infections in Taizhou, China. Methods: From 2021 to 2023, we collected 1126 plasma samples from newly diagnosed HIV patients in Taizhou. The HIV pol gene was amplified, and the obtained sequence was used to construct a maximum likelihood (ML) phylogenetic tree and molecular transmission network. PDR-related mutations were analyzed based on the Stanford University HIV Resistance Database. We conducted genotyping analysis and analysis of factors related to the larger clusters (≥10). Results: We successfully amplified and sequenced the pol region from 937 (83.2%, 937/1126) treatment-naïve HIV-1 patients, each with comprehensive epidemiological documentation. Phylogenetic characterization revealed significant subtype heterogeneity, with CRF07_BC (42.1%, 395/937), CRF01_AE (27.6%, 259/937) and CRF08_BC (22.1%, 209/937) being the most prevalent. Notably, 11.4% of the sequenced population (107/937) presented detectable PDR mutations. Univariate analysis revealed that larger clusters (≥10) are more inclined to be aged ≥60, divorced or widowed, have high or technical secondary school education, and have sexual contact with homosexuality. Multivariate analysis revealed that age ≥60 years and not having a PDR mutation (*p* < 0.05) were factors associated with larger clusters (≥10). Conclusions: Molecular transmission networks suggest that CRF08_BC is spreading rapidly among the older male population. Consequently, targeted interventions aimed at this population are crucial for halting the ongoing rapid dissemination of this subtype.

## 1. Introduction

Globally, in 2023, an estimated 30.7 million (77%) of the 39.9 million people living with HIV were on antiretroviral therapy (ART) [1]. While ART effectively suppresses viral replication [2,3], it remains noncurative and requires lifelong adherence. The expanding use of both ART and preexposure prophylaxis (PrEP) has increased concerns about increasing drug resistance [4,5]. Emerging resistance patterns threaten treatment efficacy and may facilitate transmission, particularly among high-risk populations with pretreatment resistance.

HIV exhibits high variability, yet the viral gene sequences of individuals with transmittable HIV infections show genetic similarity, tending to cluster in phylogenetic analyses [6]. As genome sequencing technology rapidly advances and HIV resistance data grow more extensive, HIV molecular transmission networks, built on viral sequences, can uncover hidden transmission links that evade detection in social networks, unhampered by social biases. Moreover, molecular transmission networks offer considerable advantages in tracking the growth of HIV transmission clusters, pinpointing risk factors for HIV transmission, and identifying individuals at risk for HIV infection [7,8].

Taizhou is located in the eastern coastal region of China and covers an area of 10,050.43 km^2^. This study is a cross-sectional analysis of newly identified HIV-1-infected individuals in the Taizhou area from 2021–2023. Through the development of a molecular transmission network and examination of pretreatment resistance gene profiles, targeted intervention strategies and optimized resource allocation can be formulated, thereby reducing the spread of HIV in Taizhou and providing foundational medication guidance for the treatment of infected individuals.

## 2. Materials and Methods

### 2.1. Study Subjects

Between 1 January 2021 and 31 December 2023, 1126 ART-naïve individuals newly diagnosed with HIV in Taizhou participated in this study. The study was reviewed and approved by the Ethics Committee of Taizhou Municipal Center for Disease Control and Prevention (Approval number: 2024012; Approval date: 9 August 2024). After providing written informed consent, the demographic information (sex, age, marital status, education level, HIV infection route) and baseline CD4^+^ T lymphocyte cell counts were collected. Blood samples were also drawn for HIV sequencing and related downstream analysis. All direct identifiers (name, address, telephone number, etc.) were removed before data analysis. During and after data collection the authors had no access to information that could identify individual participants.

### 2.2. Laboratory Methods

The preservation and initial processing of patient blood samples were performed at the Taizhou CDC. Viral RNA was subsequently isolated from plasma samples via the Tianlong RNA Extraction Kit. The extracted RNA was amplified through a two-stage polymerase chain reaction (PCR) process. Reverse transcription polymerase chain reaction (RT-PCR) for cDNA synthesis was subsequently performed via nested PCR specifically optimized to target and amplify a defined segment of the HIV-1 pol gene (corresponding to nucleotide positions 2147–3462 within the HXB2 reference strain) [9]. The amplified products were submitted to Hangzhou Qingke Zixi Biotechnology Co., Ltd. (Hangzhou, China), for electrophoretic validation, purification, and bidirectional Sanger sequencing.

### 2.3. Subtyping and HIV Drug Resistance Analysis

Consensus sequences were assembled from the sequencing results via Sequencher v5.4.6. Sequence alignment and quality control were performed via BioEdit 7.0.9. A maximum likelihood (ML) phylogenetic tree was constructed from the high-quality sequences with IQ-TREE v2.3.6 under the GTR+G+I nucleotide substitution model, with nodal support evaluated by 1000 bootstrap replicates via the Shimodaira-Hasegawa test. Reference sequences for quality validation and phylogenetic reconstruction were obtained from the Los Alamos HIV Database (https://hiv.lanl.gov; accessed on 6 September 2025). The resulting phylogenetic trees were visualized and annotated via the Interactive Tree of Life platform (iTOL v6.0; https://itol.embl.de). Pretreatment drug resistance mutations in HIV-1 polymerase sequences were analyzed via the Stanford University HIV Drug Resistance Database (https://hivdb.stanford.edu). This study only conducted genotypic resistance analysis and did not perform phenotypic resistance testing; the impact of mutations on drug susceptibility levels was automatically inferred by Stanford University HIV Drug Resistance Database without additional verification.

### 2.4. Transmission Cluster Construction

The Tamura-Nei 93 model within MEGA v6.06 was employed to calculate pairwise genetic distances (GDs). In order to improve the sensitivity of monitoring, the sequences were stratified by subtype (CRF07_BC, CRF01_AE, CRF08_BC, and other subtypes), with genetic distance thresholds determined through systematic iteration from 0.001 to 0.020 (increment = 0.001) to maximize molecular cluster formation. Molecular transmission networks were reconstructed in Cytoscape v3.9.1 by integrating sequence-derived clusters with corresponding epidemiological metadata. Consistent with established methodologies, clusters containing 2–9 cases were classified as small clusters (SCs), whereas those comprising ≥10 cases were designated as large clusters (LCs) [10].

### 2.5. Statistical Analysis

Statistical analyses were performed via R v4.4.1. Categorical epidemiological variables are summarized as frequencies and percentages. Subtype differences in demographic characteristics were assessed via χ^2^ tests. Factors associated with large cluster (≥10 cases) formation were evaluated through univariate logistic regression, with variables exhibiting *p* < 0.1 included in subsequent multivariate modeling. All tests were two-sided, with statistical significance defined as *p* < 0.05.

## 3. Results

### 3.1. Patient Demographic Characteristics

Our study obtained 937 eligible HIV-1 sequences from newly diagnosed individuals (2021–2023). As summarized in Table 1, patients were predominantly male (81.2%, 761/937), married (47.2%, 442/937), and aged 31–59 years (53.0%, 497/937). Most had primary-level education or below (44.9%, 421/937), acquired HIV through heterosexual contact (65.3%, 612/937), and presented CD4^+^ counts of 200–500 cells/mm^3^ (51.0%, 478/937). Subtype distributions differed significantly by sex, age, marital status, education level, and transmission route (*p* < 0.001).

### 3.2. HIV Subtypes

**Figure 1:**
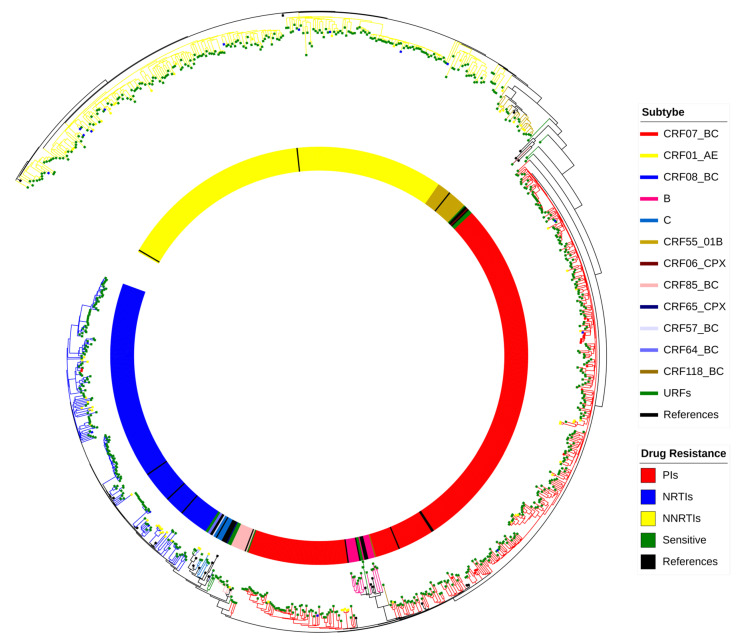
Maximum likelihood phylogenetic tree of HIV-1 pol sequences from newly diagnosed infections in Taizhou (2021–2023). The color-coded clades denote distinct subtypes, and the node colors indicate drug resistance profiles.

Twelve distinct HIV-1 subtypes along with several unique recombinant forms (URFs) were definitively identified within the 937 pol sequences. CRF07_BC emerged as the predominant subtype, constituting 42.2% (395/937) of the analyzed sequences, followed by CRF01_AE (27.6%, 259/937) and CRF08_BC (22.3%, 209/937). The other subtypes detected were CRF55_01B (2.6%, 24/937), B (1.5%, 14/937), CRF85_BC (1.3%, 12/937), C (0.6%, 6/937), CRF64_BC (0.2%, 2/937), CRF65_CPX (0.1%, 1/937), CRF57_BC (0.1%, 1/937), CRF06_CPX (0.1%, 1/937), CRF118_BC (0.1%, 1/937), and URFs (1.3%,12/937) (Figure 1).

### 3.3. Characteristics of the HIV Molecular Transmission Network

**Figure 2:**
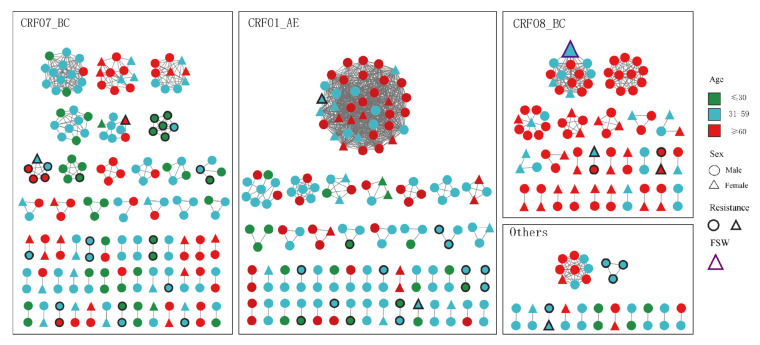
Molecular transmission networks for newly diagnosed cases, stratified by subtype. Node color indicates age, shape denotes sex, and thick borders identify individuals with drug resistance. FSWs: female sex workers.

Through GD threshold analysis, CRF07_BC, CRF01_AE, CRF08_BC and other subtypes can generate the largest number of molecular transmission networks at GD thresholds of 1.3%, 1.9%, 0.5%, and 2.0%, respectively. Classified by subtype, the molecular transmission network formed 143 clusters encompassing 465 cases (49.6%), comprising 137 small clusters and 6 large clusters. Within the CRF07_BC network, 183 samples connected, forming 58 clusters (55 SCs; 3 LCs); the CRF01_AE network contained 159 samples, constituting 46 clusters (45 SCs; 1 LCs); and CRF08_BC included 89 samples, forming 26 clusters (24 SCs; 2 LCs). In addition, the other subtypes generated 13 small clusters with 34 connected samples (Figure 2).

### 3.4. Characterization of Large Clusters

Our analysis identified six large transmission clusters. The largest cluster (LC1, CRF01_AE) comprised 38 cases. CRF07_BC contained three large clusters: LC2 (n = 14), LC4 (n = 11), and LC6 (n = 10). Similarly, CRF08_BC formed two large clusters: LC3 (n = 14) and LC5 (n = 11). Longitudinal tracking (2021–2023) revealed ≥2 new cases incorporated annually into each cluster. Within these clusters (total n = 98), patients aged ≥60 years accounted for 71.4% (70/98), and males constituted 86.7% (85/98). Heterosexual transmission represented 83.7% (82/98) of the cases, whereas 78.6% (77/98) were registered residents of Taizhou. The detailed characteristics are presented in Table 2. Notably, only one pretreatment drug resistance (PDR) case was observed, exclusively in LC1.

Univariate analysis revealed significant associations between large cluster formation (≥10 cases) and the following factors (*p* < 0.001): age ≥ 60 years, divorced/widowed marital status, high school/technical secondary education, and homosexual contact. Multivariate analysis further revealed that patients aged ≥60 years without PDR mutation were independently associated with inclusion in large molecular transmission clusters (*p* < 0.05) (Table 3).

### 3.5. PDR Distribution and Drug Resistance Mutation Analysis

Pretreatment drug resistance (PDR) mutations were detected in 107 patients (11.4%, 107/937). Nonnucleoside reverse transcriptase inhibitor (NNRTI) resistance occurred in 7.7% (72/937), nucleoside reverse transcriptase inhibitor (NRTI) resistance occurred in 1.9% (18/937), and protease inhibitor (PI) resistance occurred in 1.8% (17/937) (Figure 1).

**Figure 3:**
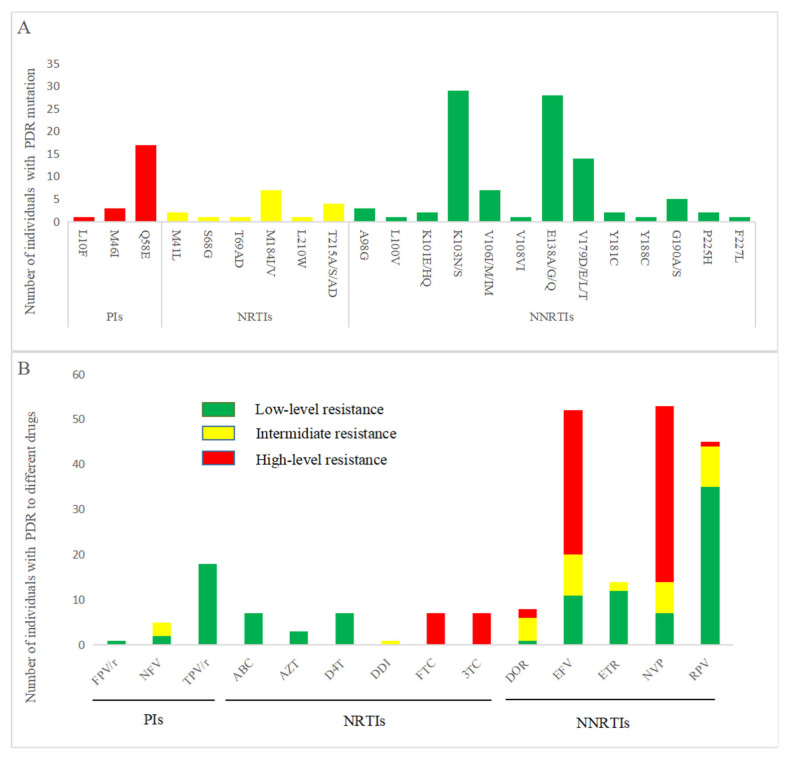
(**A**). Pretreatment number of drug resistance (PDR) to major drug classes. (**B**). HIV-1 PDR mutation levels in response to ART. PI: Protease inhibitor; NRTI: Nucleoside reverse transcriptase inhibitor; NNRTI: Nonnucleoside reverse transcriptase inhibitor; FPV/r: Fosamprenavir/ritonavir; NFV: Nelfinavir; TPV/r: Ritonavir/ritonavir; ABC: Abacavir; AZT: Zidovudine; D4T: Stavudine; DDI: Didanosine; FTC: Emtricitabine; 3TC: Lamivudine; DOR: Doravirine; EFV: Efavirenz; ETR: Etravirine; NVP: Nevirapine; RPV: Rilpivirine.

We identified 4 PI-associated, 7 NRTI-associated, and 22 NNRTI-associated resistance mutation sites. The most prevalent mutation was NNRTI-associated K103N (21.5%, 23/107), which conferred high-level resistance to nevirapine (NVP) and efavirenz (EFV) in the highest proportion of affected patients. Among the PI-associated mutations, Q58E was the most common (15.9%, 17/107), primarily causing low-level resistance to tipranavir (Figure 3).

## 4. Discussion

Molecular transmission networks utilizing the HIV-1 pol gene enable targeted interventions against HIV transmission [11,12,13,14,15]. By conducting phylogenetic analysis on the basis of virus sequences, we can establish HIV transmission networks among relevant individuals to map transmission dynamics, pinpoint spread sources, and identify active networks enabling precise interventions for high-risk carriers. Through a 2021–2023 cross-sectional study of newly diagnosed HIV-1 patients in Taizhou, eastern China, we integrated molecular epidemiology data to construct a city transmission network.

This study revealed that sex, age, educational level, marital status, and infection route all have significant effects on the distribution of subtypes in the Taizhou area. Compared to Hangzhou and Nanjing [9,10], six predominant subtypes or CRFs and at least six more less predominant HIV lineages are circulating in Taizhou, demonstrating greater HIV-1 genetic diversity and epidemic complexity in Taizhou. Furthermore, unlike other cities in Zhejiang Province [16,17,18], CRF08_BC has become the third major subtype in Taizhou, following CRF07_BC and CRF01_AE. Through gene threshold screening, we discovered that the gene threshold for CRF08_BC stands at 0.5%, which is notably lower than the 1.3% for CRF07_BC and the 1.9% for CRF01_AE. These findings suggest that CRF08_BC has tended to spread more rapidly in recent years in Taizhou. In addition, among the six LCs, 71.4% (70/98) were over 60 years old, with males accounting for 86.7% (85/98) and heterosexual transmission accounting for 83.7% (82/98) of the LCs. Moreover, this phenomenon is particularly pronounced within the two LCs of CRF08_BC, as evidenced by Table 2 (≥60, 72%; males, 88%; heterosexual transmission, 96%). Through epidemiological investigations, we discovered a female sex worker in LC3 with household registration in Deyang city. Geographically, she is located at the border of Yunnan Province, which is the main epidemic area [19,20,21,22] for CRF08_BC. This, to some extent, indicates that CRF08_BC was introduced into Taizhou through heterosexual transmission, increasing the subtype diversity in Taizhou. These findings suggest that males in this age group are the primary drivers of HIV transmission through heterosexual transmission in Taizhou.

The overall prevalence of pretreatment drug resistance (PDR) in our study was 11.4%, which was classified as moderate (5–15%) according to the WHO HIV drug resistance thresholds [23]. The most frequently detected NNRTI-associated mutation, K103N, confers resistance to efavirenz (EFV) and nevirapine (NVP), which aligns with prior reports [24,25]. Since 2004, China’s national ART program has provided EFV- or NVP-based first-line regimens free of charge. The prolonged utilization of these NNRTIs has driven substantial accumulation of resistance-conferring mutations in treated populations, consequently increasing the incidence of NNRTI-associated PDR among newly diagnosed individuals. Among the PI-associated mutations, Q58E had the highest frequency, which is consistent with the findings of multiple studies [26,27,28] and reduces susceptibility to tipranavir/ritonavir (TPV/r). However, as TPV/r remains nonformulary in China’s free treatment program and is rarely used in the clinic [29,30], the use of Q58E has a limited impact on the selection of ART regimens for PDR-affected patients. Overall, the situation of drug resistance mutations in Taizhou is at a normal level in Zhejiang Province, and the molecular transmission network also indicates that PDR is not a key transmission factor. Nevertheless, developing tailored therapeutic strategies for first-line ART failure remains essential, alongside sustained surveillance of PDR-associated molecular clusters. Failure to implement these measures risks compromising treatment efficacy and accelerating PDR dissemination within transmission networks.

We found that the overall transmission of drug resistance is not expected to negatively impact current antiviral treatment strategies in the near future; however, because of the generally lower educational attainment of individuals aged ≥60 years, their primary sources for acquiring knowledge about AIDS prevention and control are television, radio, posters, and brochures. These individuals often lack knowledge about AIDS prevention and maintain a certain sexual demand, leading them to predominantly seek services from low-priced female sex workers [31,32]. However, female sex workers offering low prices often have lower condom use rates and a greater risk of AIDS infection and transmission. Furthermore, the poor awareness of disease prevention among elderly people exacerbates the risk of infection. Therefore, health departments should strengthen the publicity and intervention of key female sex worker activity sites, carry out AIDS screening for elderly individuals in combination with health examinations, increase their awareness of sexual behavior safety, detect cases in time, and reduce the spread of AIDS.

This study has several limitations. First, we detected four patterns of protease inhibitor (PI) resistance-related mutations but did not further investigate their sources. Second, this study only sequenced the pol region but not the gag region, which may lead to an underestimation of resistance related to protease inhibitors [33]. Moreover, since this study was limited to genotypic resistance analysis and did not include phenotypic resistance testing, we were unable to follow up and evaluate the virological suppression of all participants, as has been done in related studies [34]. Furthermore, the molecular transmission networks were constructed on the basis of the available case samples, inevitably excluding untested or undiagnosed individuals who may contribute to ongoing transmission. Therefore, other molecular clusters have not been able to trace possible sources of infection due to the dark flow of positive cases.

## 5. Conclusions

Our study demonstrated the disproportionate contribution of older adults to HIV transmission dynamics in Taizhou, particularly within CRF08_BC molecular clusters, as evidenced by molecular network analysis. The LC3 cluster further substantiates the role of female sex workers as critical transmission conduits bridging infection networks among this aging demographic. These findings underscore the imperative for sustained genetic surveillance of incident HIV cases, targeted public health initiatives enhancing geriatric HIV/AIDS literacy, and tailored structural interventions disrupting transmission pathways in high-risk elderly populations.

## Figures and Tables

**Table 1:** Sociodemographic characteristics of newly diagnosed HIV-1 patients in Taizhou from 2021–2023.

	Cases (%) (n = 937)	HIV Subtypes (%)				χ^2^	*p*
		CRF07_BC (n = 395)	CRF01_AE (n = 259)	CRF08_BC (n = 209)	Others (n = 74)		
Collection time						10.337	0.111
2021	344 (36.7)	150 (38.0)	92 (35.5)	67 (32.1)	35 (47.3)		
2022	289 (30.8)	114 (28.9)	85 (32.8)	65 (31.1)	25 (33.8)		
2023	304 (32.4)	131 (33.2)	82 (31.7)	77 (36.8)	14 (18.9)		
Sex						23.381	*p* < 0.001
Male	761 (81.2)	335 (84.8)	220 (84.9)	146 (69.9)	60 (81.1)		
Female	176 (18.8)	60 (15.2)	39 (15.1)	63 (30.1)	14 (18.9)		
Age (years)						99.917	*p* < 0.001
≤30	155 (16.5)	97 (24.6)	40 (15.4)	7 (3.3)	11 (14.9)		
31–59	497 (53.0)	210 (53.2)	154 (59.5)	87 (41.6)	46 (62.2)		
≥60	285 (30.4)	88 (22.3)	65 (25.1)	115 (55.0)	17 (23.0)		
Married status						68.190	*p* < 0.001
Singal	272 (29.0)	152 (38.5)	82 (31.7)	18 (8.6)	20 (27.0)		
Married	442 (47.2)	173 (43.8)	103 (39.8)	128 (61.2)	38 (51.4)		
Divorced/Widowed	223 (23.8)	70 (17.7)	74 (28.6)	63 (30.1)	16 (21.6)		
Education						122.43	*p* < 0.001
Primary school/illiterate	421 (44.9)	130 (32.9)	106 (40.9)	158 (75.6)	27 (36.5)		
Junior middle school	275 (29.3)	129 (32.7)	79 (30.5)	37 (17.7)	30 (40.5)		
High or technical secondary school	147 (15.7)	74 (18.7)	47 (18.1)	12 (5.7)	14 (18.9)		
Junior college or above	94 (10.0)	62 (15.7)	27 (10.4)	2 (1.0)	3 (4.1)		
Sexual contact						112.59	*p* < 0.001
Heterosexual	612 (65.3)	215 (54.4)	149 (57.5)	198 (94.7)	50 (67.6)		
Homosexual	320 (34.2)	179 (45.3)	108 (41.7)	9 (4.3)	24 (32.4)		
Others	5 (0.5)	1 (0.3)	2 (0.8)	2 (1.0)	0 (0.0)		
CD4^+^ T lymphocyte count						10.895	0.092
<200	378 (40.3)	140 (35.4)	121 (46.7)	81 (38.8)	36 (48.6)		
200–499	478 (51.0)	220 (55.7)	117 (45.2)	109 (52.2)	32 (43.2)		
≥500	81 (8.6)	35 (8.9)	21 (8.1)	19 (9.1)	6 (8.1)		

**Table 2:** Summary statistics of the six large clusters in Taizhou city from 2021–2023.

LC	Subtype	Nodes	Growing Case (%)			Age ≥ 60 (%)	Male (%)	Heterosexual (%)	Taizhou Household Registration (%)
			2021	2022	2023				
LC1	CRF01_AE	38	16 (42.1)	8 (21.1)	14 (36.8)	25 (65.7)	26 (68.4)	35 (92.1)	29 (76.3)
LC2	CRF07_BC	14	6 (42.8)	4 (28.6)	4 (28.6)	14 (40.0)	26 (74.2)	2 (14.2)	7 (50.0)
LC3	CRF08_BC	14	1 (7.2)	5 (35.7)	8 (57.1)	7 (50)	11 (78.5)	14 (100.0)	10 (71.4)
LC4	CRF07_BC	11	4 (36.4)	2 (18.2)	5 (45.4)	8 (72.7)	5 (54.5)	11 (100.0)	10 (90.9)
LC5	CRF08_BC	11	2 (18.2)	5 (45.4)	4 (36.4)	11 (100.0)	11 (100.0)	10 (90.9)	11 (100.0)
LC6	CRF07_BC	10	5 (50.0)	3 (30.0)	2 (20.0)	5 (50.0)	6 (60.0)	10 (100.0)	10 (100.0)

**Table 3:** Factors associated with large-molecule transmission cluster membership in Taizhou, China, 2021–2023.

	Total N (%) ^a^	Not Included in a Large Cluster (<10) N (%) ^b^	Included in a Large Cluster (≥10) N (%) ^b^	Univariate Analysis		Multivariate Analysis	
				OR (95CI)	*p*	AOR (95CI)	*p*
HIV subtypes							
CRF07_BC	395 (42.2)	360 (42.9)	35 (35.7)				
CRF01_AE	259 (27.6)	221 (26.3)	38 (38.8)	1.77(1.08–2.88)	0.022	1.50 (0.89–2.53)	*p* = 0.130
CRF08_BC	209 (22.3)	184 (21.9)	25 (25.5)	1.40(0.81–2.41)	0.227	0.67 (0.37–1.20)	*p* = 0.177
Others	74 (7.9)	74 (8.8)	0 (0.0)	0	0.974	0	*p* = 0.988
Collection time							
2021	344 (36.7)	310 (36.9)	34 (34.7)				
2022	289 (30.8)	262 (31.2)	27 (27.6)	0.94 (0.55–1.60)	*p* = 0.818		
2023	304 (32.4)	267 (31.8)	37 (37.8)	1.26 (0.77–2.07)	*p* = 0.353		
Gender							
Male	761 (81.2)	687 (81.9)	74 (75.5)				
Female	176 (18.8)	152 (18.1)	24 (24.5)	1.47 (0.90–2.40)	*p* = 0.128		
Age (years)							
≤30	155 (16.5)	153 (18.2)	2 (2.0)				
31–59	497 (53.0)	458 (54.6)	39 (39.8)	6.51 (1.55–27.30)	*p* = 0.010	3.21 (0.67–15.43)	*p* = 0.145
≥60	285 (30.4)	228 (27.2)	57 (58.2)	19.12 (4.60–79.50)	*p* < 0.001	6.91 (1.34–35.53)	*p* = 0.021
Married status							
Singal	272 (29.0)	261 (31.1)	11 (11.2)				
Married	442 (47.2)	392 (46.7)	50 (51.0)	3.03 (1.55–5.92)	*p* = 0.001	0.82 (0.37–1.82)	*p* = 0.632
Divorced/Widowed	223 (23.8)	186 (22.2)	37 (37.8)	4.72 (2.35–9.49)	*p* < 0.001	1.25 (0.55–2.81)	*p* = 0.597
Education							
Primary school/illiterate	421 (44.9)	350 (41.7)	71 (72.4)				
Junior middle school	275 (29.3)	256 (30.5)	19 (19.4)	0.37 (0.22–0.62)	*p* < 0.001	0.60 (0.33–1.08)	*p* = 0.087
High or technical secondary school	147 (15.7)	139 (16.6)	8 (8.2)	0.28 (0.13–0.60)	*p* = 0.001	0.62 (0.27–1.46)	*p* = 0.277
Junior college or above	94 (10.0)	94 (11.2)	0 (0.0)	0	*p* = 0.980	0	*p* = 0.987
Sexual contact							
Heterosexual	612 (65.3)	530 (63.2)	82 (83.7)				
Homosexual	320 (34.2)	304 (36.2)	16 (16.3)	0.34 (0.20–0.59)	*p* < 0.001	0.61 (0.32–1.14)	*p* = 0.122
Others	5 (0.5)	5 (0.6)	0 (0.0)	0	*p* = 0.983	0	*p* = 0.997
CD4^+^ T lymphocyte count							
<200	378 (40.3)	333 (39.7)	45 (45.9)				
200–499	478 (51.0)	433 (51.6)	45 (45.9)	0.77 (0.50–1.19)	*p* = 0.239		
≥500	81 (8.6)	73 (8.7)	8 (8.2)	0.81 (0.37–1.79)	*p* = 0.605		
PDR mutation							
Yes		106 (12.6)	1 (1.0)				
No		733 (87.4)	97 (99.0)	14.03 (1.94–101.65)	*p* = 0.009	10.16 (1.38–74.75)	*p* = 0.023

Parentheses denote ^a^ overall subject proportion (%) per variable and ^b^ cluster member proportion (%) per subgroup.

## Data Availability

The datasets used and/or analyzed during this study are available from the corresponding author upon reasonable request and with permission from the Taizhou Municipal Center for Disease Control and Prevention, Taizhou Municipal Institute of Health Supervision.

## Abbreviations

HIV	Human immunodeficiency virus
AIDS	Acquired immunodeficiency syndrome
ART	Antiretroviral therapy
WHO	World Health Organization
PDR	Pretreatment drug resistance
PCR	Polymerase chain reaction
PI	Protease inhibitor
NRTI	Nucleoside reverse transcriptase inhibitor
NNRTI	Nonnucleoside reverse transcriptase inhibitor
FPV/r	Fosamprenavir/ritonavir
NFV	Nelfinavir
TPV/r	Ritonavir/ritonavir
ABC	Abacavir
AZT	Zidovudine
D4T	Stavudine
DDI	Didanosine
FTC	Emtricitabine
3TC	Lamivudine
DOR	Doravirine
EFV	Efavirenz
ETR	Etravirine
NVP	Nevirapine
RPV	Rilpivirine
